# Internet-based cognitive-behavioral therapy is effective in reducing depressive symptomatology in type 1 diabetes: results of a randomized controlled trial

**DOI:** 10.3389/fcdhc.2023.1209236

**Published:** 2023-11-07

**Authors:** Mónica Carreira, Ma Soledad Ruiz de Adana, José Luis Pinzón, María Teresa Anarte-Ortiz

**Affiliations:** ^1^ Department of Personality, Assessment and Psychological Treatment, Instituto de Investigación Biomedica de Málaga (IBIMA), University of Malaga, Malaga, Spain; ^2^ Clinical Management Unit of Endocrinology and Nutrition, Instituto de Investigación Biomedica de Málaga (IBIMA), Regional University Hospital, University of Malaga, Malaga, Spain; ^3^ Clinical Management Unit of Endocrinology and Nutrition, Instituto de Investigación Biomedica de Málaga (IBIMA), Virgen de la Victoria University Hospital, University of Malaga, Malaga, Spain

**Keywords:** depressive symptomatology, type 1 diabetes, internet-based, cognitive-behavioral therapy (CBT), efficacy

## Abstract

**Objective:**

Depression in people with diabetes is associated with poorer health outcomes. Although web programs integrating cognitive-behavioral therapy with diabetes education have shown good results, no similar approach has been implemented in Spain. This aim of this study was to administer an Internet-based cognitive-behavioral therapy program (CBT) for the treatment of mild-moderate depressive symptomatology in individuals with type 1 diabetes (WEB_TDDI1 study) and evaluate the efficacy of this program.

**Research design and methods:**

A pre-post randomized controlled study was conducted. The sample comprised 65 people with type 1 diabetes and mild-moderate depressive symptoms: 35 treatment group (TG) and 30 control group (CG). The following effects of the nine-session program were analyzed: depression (Beck Depression Inventory Fast Screen, BDI-FS), metabolic variables (glycosilated hemoglobin, HbA1c), and other psychological variables including anxiety (State Trait Anxiety Inventory, STAI), fear of hypoglycemia (Fear of Hypoglycemia Questionnaire, FH-15), distress (Diabetes Distress Questionnaire (DDS), quality of life (Diabetes Quality of Life Questionnaire, DQOL),and treatment adherence (Diabetes Self-Care Inventory-Revised questionnaire, SCI-R).

**Results:**

At the end of the treatment program, only 28 people were evaluated (TG=8; CG=20). However, a significant reduction was found in both groups in BDI-FS and STAI-T scores, which was significantly greater in the TG. Significant improvements were also found in the TG in DQOL, FH-15, DDS and SCI-R scores. The percentage change in these variables was also statistically significant in the TG versus the CG. However, no significant results were found in HbA1c.

**Conclusions:**

The Internet-based cognitive-behavioral therapy program for the treatment of mild-moderate depressive symptomatology in people with type 1 diabetes (WEB_TDDI1 study) is effective in reducing depressive symptomatology in the sample that completed the study. Positive results are also produced in other variables associated with depression in this population such as diabetes-related distress, trait anxiety, fear of hypoglycemia, quality of life, and adherence to diabetes treatment. Although new studies would be necessary to support the results of this platform, the results obtained are positive and support the use of this platform as an appropriate treatment for this population.

**Clinical trial registration:**

ClinicalTrials.gov; identifier NCT03473704.

## Highlights

- The program designed in the WEB_TDD1 study produces improvements in depressive symptomatology in individuals with type 1 diabetes.
- This program produces improvements in other variables associated with depression in diabetes such as anxiety, diabetes-related distress, fear of hypoglycemia and treatment adherence.
- This program is designed to be carried out online.

## Introduction

Individuals with type 1 diabetes have a higher prevalence of depression than the general population (1, 2), with important negative consequences in their daily lives associated with an increased risk of microvascular and macrovascular complications (3). In addition, having type 1 diabetes and depression is associated with poorer treatment adherence (4), poorer quality of life (5), and higher costs (6, 7) than having diabetes without depression. It is therefore necessary to identify and appropriately treat depression.

Research has confirmed the effectiveness of treatment of depression and depressive symptomatology in people with diabetes, with the effect of intervention found to be superior to usual treatment, placebo, waiting list, or comparative treatment in clinical trials (8). Among the interventions that have been evaluated are group therapy, online treatment, exercise, pharmacological treatment, psychotherapy, collaborative care, and telephone treatment. Cognitive-behavioral therapy (CBT) is one of the psychological interventions used,as it is an effective treatment for adults with depression (9). In the population with diabetes, four meta-analyses of randomized controlled trials (10–13) and one systematic review (14) indicate that CBT is an appropriate treatment for depression.

New technologies have proven to be a good tool for the treatment of depression, facilitating accessibility and reducing the stigma associated with the disease (15). The recent COVID-19 pandemic highlighted the need to design telematic tools for psychological treatment of the population, with contributions made in the field of diabetes (16). The results of Internet-based depression treatments in diabetes have shown positive results in reducing depressive symptomatology, but no significant results have been found in glycemic control (8, 17).

In this vein, several authors have designed web programs incorporating CBT for people with diabetes. Pioneers in this area, van Bastelaar et al. (18–20) designed and administered a specific treatment program for people with diabetes that comprised eight sessions, comparing the results with those of a control group that received no treatment. They obtained positive results in depression and specific distress but not in glycemic control. Based on the work of van Bastelaar et al. (18–20), Nobis et al. (21–23) developed a web-based intervention with telephone support for adults with diabetes. In this case, the results of the six-session web treatment (plus two optional sessions) were compared with a psychoeducation program. Again, there was an improvement in depression and diabetes-specific distress in the intervention group, but there were no changes in glycemic control. The improvements found were maintained at the 6-month follow-up. Other authors have assessed the effectiveness of web-based CBT treatment programs for the general population in individuals with diabetes. Robins et al. (24) designed a study comparing a six-session web-based treatment for the general population with a control group treated with usual treatment. Positive results were obtained in mood and diabetes-specific distress, anxiety, and on the mental health quality of life scale of the SF-12, although not on the physical scale. No significant results were found in glycemic control. These results were maintained at the 3-month follow-up (25). Clarke et al. (26) designed a study to evaluate the effect of a general population CBT program (myCompass) on the well-being of young people with type 1 diabetes. The results of this study have not yet been published. Nevertheless, they analyzed the results of this seven-session program in a pre-post study without a control group in a population with type 1 and type 2 diabetes and found significant improvement in depressive symptoms and specific distress and anxiety (moderate effect size), although data on glycemic control were not collected (27).

CBT has therefore shown positive results in the population with diabetes, but its effect on other variables related to depression remains to be to be demonstrated. Despite the improvements in depressive symptoms and the need to create new tools to facilitate access to depression treatment for the population with diabetes, in Spain no such resources are available specific to this population. On the other hand, this platform has been specifically designed for people with type 1 diabetes, including specific modules to work on depressive symptoms in this population. In this sense, it is specific for people with type 1 diabetes, since the differential characteristics between type 1 diabetes and type 2 diabetes mean that they must be treated differently (28, 29). The new technological scenario derived from the COVID-19 pandemic and the advances in new technologies in the treatment of people with type 1 diabetes have meant that professionals specialized in type 1 diabetes must make new approaches that are increasingly personalized and adjusted to the needs of the people with whom they work. The use of web platforms facilitate this personalization and a more continuous contact between the doctor and the person with type 1 diabetes and, above all, it can help in the self-management of the users.

For these reasons, the main objective of this study was to administer an Internet-based CBT program designed and developed by our research group (30) for the treatment of mild-moderate depressive symptomatology in individuals with type 1 diabetes (WEB_TDDI1 study) and to evaluate the efficacy of this program.

## Research design and methods

### Participant recruitment

The sample was recruited between January 2017 and March 2019. Participants were individuals with type 1 diabetes attending the Clinical Management Unit of Endocrinology and Nutrition of two hospitals (tertiary care) and a specialty center (secondary care) of the Andalusian Health Service of the province of Malaga (Spain). The participants were informed of the study through the information given in the unit areas and/or by their physician. Those who wished to participate voluntarily were evaluated by a psychologist from our research team to determine whether they met the inclusion/exclusion criteria.The inclusion criteria were: medical diagnosis of type 1 diabetes ≥1 year; over 18 years of age; mild-moderate depressive symptoms; no concomitant pharmacological treatment that could modify blood glucose values or depressive symptomatology; no psychological treatment at the present time; absence of the following: chronic renal failure, impaired liver function tests, active thyroid disease (except correctly substituted hypothyroidism), current pregnancy, or acute ketosis decompensation at the beginning of the study; Internet access. Exclusion criteria were: type 2 diabetes; women who were pregnant or planning to become pregnant; severe macrovascular or microvascular complications; no depressive symptoms; severe depressive symptoms; diagnosis of major depressive disorder with risk of suicide; non-collaboration (no signed informed consent); presenting a disabling psychiatric disorder; psychosis; no Internet access.Those participants who met the inclusion criteria were evaluated and randomized through a computer program (SPSS), carry out said randomization to 50% of the participants to each group, to one of the two study groups: the treatment group (TG),which received the 9-week web-based treatment, or the control group (CG) in which the participants were on a waiting list. The procedure for calculating the sample size is described in Carreira et al. (30). Having a power of 0.80 and a significance level of 0.05, a sample size of 28 participants in each group is necessary for an effect size of 0.50. In this sense, the starting point was the need for a total of 56 participants for the present study. All the people included in the study signed the informed consent.

### Description of intervention

The participants assigned to the TG received an email providing the link to the web page where the treatment program was hosted together with their username and password (modifiable by the user). The web program includes nine weekly sessions based on CBT. The topics were: Depression. Diabetes and depression, Stress and diabetes, Coping in diabetes, Resolution of problems, Pleasurable activities, Cognitive restructuring, Social skills, Importance of support, relapse prevention (30).

**Figure 1:**
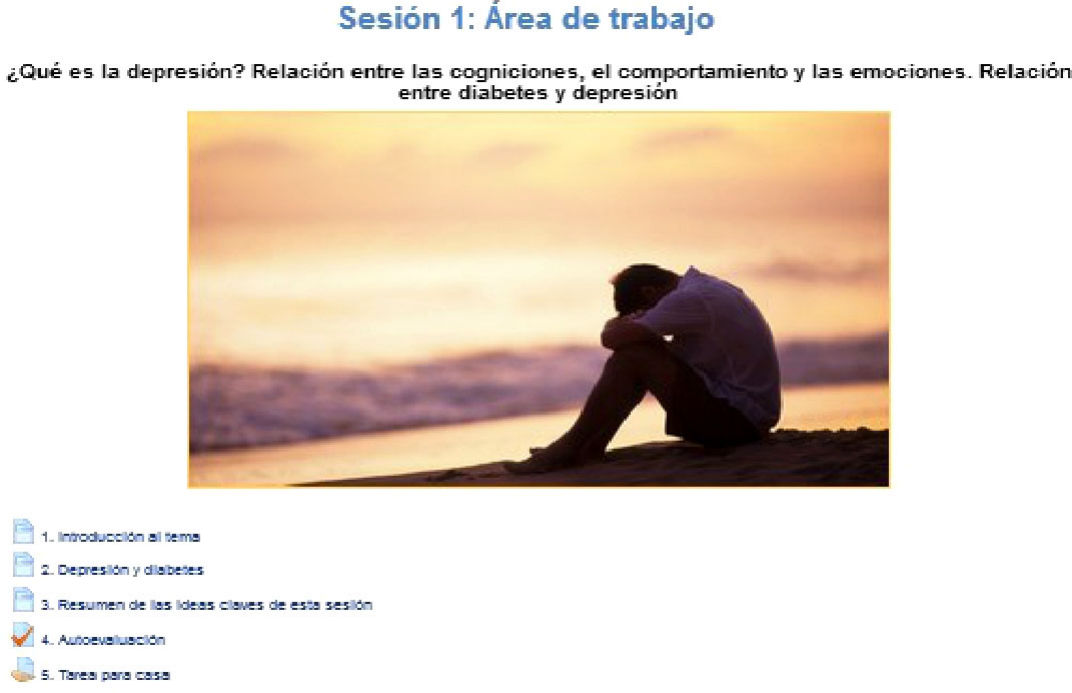
Screenshot of the initial outline of a session.

In each session, as can be seen in **Figure 1**, the topic to be worked on was addressed with information and examples, as well as a summary of the main key ideas and self-assessment of the knowledge acquired in the session. After this, the self-work task for the week was explained to the participant with an example of the task. Once the activity was completed and sent by the user to the web page, the therapist provided brief feedback and possible recommendations. To continue with the next session, the current task had to be completed. The duration of each session was approximately 20-30 minutes.

Halfway through the program, the participants were asked to provide feedback on the sessions and program topics. Participants who did not adhere to the program were emailed or phoned to find out why and to motivate them to continue. The CG participants were on a waiting list until the TG participants finished the program. Those who still met the inclusion criteria at the end of their participation in the CG program were enrolled in the treatment program.

### Study design

The study design was a pre-post randomized controlled study. In the post-assessment, participants were evaluated upon completing the 9-session program. This article follows the CONSORT reporting guidelines (31). This study was approved by the Provincial Research Ethics Committee of Malaga (approval number: 1216/PIA14), Spain (December 20, 2016).

### Hypothesis

It was anticipated that the Internet-based program for the treatment of mild-moderate depressive symptomatology in individuals with type 1 diabetes (WEB_TDDI1 study) based on CBT would be effective in reducing depressive symptomatology. It was also expected that this improvement would be reflected in the other psychological variables evaluated. Consequently, the efficacy of this tool for the treatment of depressive symptomatology in people with type 1 diabetes was expected to be demonstrated.

### Outcome measures

A structured interview was used to collect sociodemographic data, as well as healthy habits, medication being taken, and toxic habits. Any previous mental health treatment and diabetes-related support was also recorded.

Clinical data and glycemic control, analyzed with the glycosylated hemoglobin (HbA1c) test, were collected through a structured interview from the data recorded in the medical review from the information provided by the physician and the participant. The HbA1c values were collected by the health professionals at the two points of the evaluation of the participants (baseline and post-treatment).

The structured Clinical Interview for DSM-5 Disorders-Clinician Version (32) was used to assess the presence of a major depressive episode.

#### BDI-FS

The Spanish version of the Beck Depression Inventory-Fast Screen (BDI-FS) (33) was used to assess the depressive symptomatology of the study participants. The BDI-FS evaluates the intensity of depressive symptomatology in the last two weeks in medical patients. It is a self-administered questionnaire with seven items with a score from 0 to 3 points. This questionnaire is used as a screening tool to detect mild-moderate symptomatology. For this purpose, the score on the questionnaire had to range between 4 and 12 points.

#### FH-15

Fear of hypoglycemia was assessed with the Fear of Hypoglycemia questionnaire (FH-15) (34). This instrument consists of fifteen negative items, evaluated using a Likert-type scale with values ranging from 1 to 5 (1=Never to 5 =Every day). This questionnaire has a cut-off point of 28 points that allows the identification of individuals with a fear of hypoglycemia.

### DDS

To evaluate the level of diabetes-related stress, the Diabetic Distress Questionnaire (DDS) by Polonsky et al. (35, 36) was used. The DDS has sixteen Likert-type items, with five response options from strongly disagree (1) to strongly agree (5), whereby a higher score indicates a higher level of stress. This scale yields a total distress score and four dimensions: emotional distress; doctor-patient relationship distress; regimen-related distress; and interpersonal distress.

#### DQOL

Diabetes Quality of Life Questionnaire (DQOL) (37). This questionnaire assesses quality of life specifically in individuals with diabetes mellitus. The Spanish version (38) comprises a total of forty-three questions. It has four subscales: Dissatisfaction, Impact, Social/Vocational worry and,Worry about future effects. Responses are quantified using a Likert scale with five ordinal responses.

#### STAI

The Spanish version of the State Trait Anxiety Inventory adapted by TEA (39, 40) was used to assess anxiety. The State-Trait Anxiety Inventory (STAI) comprises two self-assessment scales that measure two independent concepts of anxiety: state (S) and trait (T). The State-Anxiety scale (STAI-S) assesses how the subject feels in threatening situations at a specific time, while the Trait-Anxiety scale (STAI-T) assesses permanence of anxiety in the subject, assessing a propensity to respond with elevated rates of anxiety to stressful situations. Each scale consists of twenty self-administered items rated on a Likert scale (0–3).

#### SCI-R

The Spanish version of the Diabetes Self-Care Inventory-Revised questionnaire (SCI-R) was used to evaluate treatment adherence (41). The SCI-R is composed of fifteen questions that assess the patient’s perception of adherence to diabetes self-care recommendations in the last month. Each question is scored on a Likert scale from 1 (never) to 5 (always).

### Timing of evaluations

The baseline evaluations were conducted in person by a psychologist from the research team. At the end of the treatment program forthe TG, the study participants in both groups were evaluated (TG and CG).

### Statistical analyses

The analyses were carried out with IBM SPSS Statistics, version 18.0. First, the descriptors of the sample were analyzed by groups. The quantitative variables were described with measures of central tendency and dispersion. For the qualitative variables, a frequency analysis was performed. The relationship between the qualitative variables was evaluated with the χ2 test. Given the sample size in the different evaluations and that the variables did not meet the normality assumption, non-parametric tests were used to analyze cross-sectional differences. To contrast differences between the two groups (TG and CG) at baseline and post-treatment, the Mann-Whitney U-test was used. In addition, to analyze the possible differences between the two groups after treatment in the variables studied, the percentage change in both groups between the baseline and post-treatment values was analyzed using the following formula: [(baseline score-post-treatment score)/baselinescore]×100.

All analyses were performed according to the protocol. Analysis of the results began when the TG subjects had completed all phases of the treatment program. An improvement in the different variables wastaken into accountwhen there were significant differences in p ≤ 0.05. Hypotheses were contrasted at a 95% confidence level.

## Results

**Figure 2:**
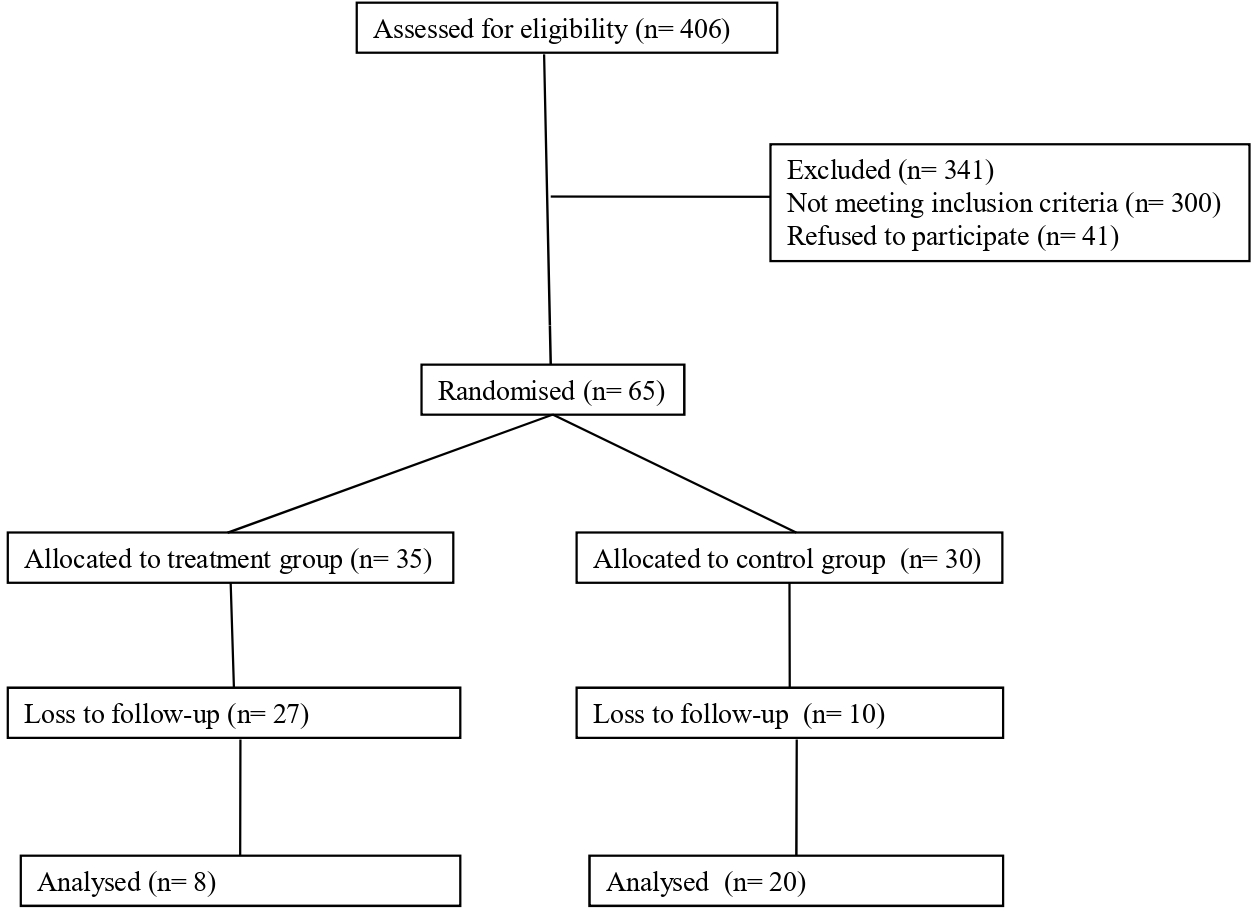
CONSORT study flowchart.

A total of 406 individuals with type 1 diabetes were evaluated during the recruitment period as potential study participants. Of these, 65 patients with type 1 diabetes (16.01%) were finally included in the study and underwent the initial evaluation (see **Figure 2**). The main reasons for non-inclusion were: not meeting any of the criteria; although they met the criteria, they did not undergo the initial evaluation; or problems with the use of new technologies (only 2.6%).

The sample was randomized and the subjects assigned to the two study groups (TG=35 and CG=30). The main characteristics of the participants are presented in **Table 1**. No significant differences were found between the two groups at baseline in the sociodemographic, clinical or health habits variables. Nor were significant differences found in the psychological variables studied between the two at baseline (**Table 2**).

**Table 1:** Main descriptors of the sample.

Variable	Treatment Group (TG) (n=35)	Control Group (CG) (n=30)	p
Age*(years), mean (SD)	37.48 (11.01)	35.03 (12.87)	0.340
Sex, n (%) Men Women	9 (25.7%) 26 (74.3%)	11 (36.7%) 19 (63.3%)	0.340
Time with diabetes* (years), mean (SD)	18.71(12.72)	21.26 (12.82)	0.437
Major depressive disorder (Yes), n (%)	6 (17.1%)	5 (16.7%)	0.959
Previous psychological or psychiatric treatment (Yes), n (%)	23 (65.7%)	14 (46.7%)	0.122
HbA1c* (%), mean (SD)	8.22 (1.51)	8.31(1.48)	0.657
Diabetes or other complications (Yes), n (%)	22 (62.9%)	16 (53.3%)	0.437
Perceived support regarding diabetes (Yes), n (%)	33 (94.3%)	28 (93.3%)	0.873
Smoker (Yes), n (%)	10 (28%)	9 (30%)	0.900
Alcohol consumption (Yes), n (%)	12(34.3%)	11 (36.7%)	0.841
Drug use (Yes), n (%)	2 (5.7%)	1 (3.3%)	0.648
Exercise (Yes), n (%)	19 (54.3%)	16 (53.3%)	0.939
Sleep well (Yes), n (%)	16 (45.7%)	14 (46.7%)	0.129

* Mean (Standard deviation).

**Table 2:** Baseline and post-treatment results in the main variables.

Variable (scores)	Treatment Group (TG)			Control Group (CG)			Between group baseline p	Between group post-treatment p
	Baseline (n=35)	Post-treatment (n=8)	p	Baseline (n=30)	Post-treatment (n=20)	p		
BDI-FS	6.54 (2.04)	0.62 (1.41)	**0.012**	6.96 (1.92)	4.60 (3.06)	**0.016**	0.315	**0.001**
DQOL_total	110.57 (18.62)	79.00 (9.81)	**0.012**	112.90 (25.21)	108.00 (25.16)	0.053	0.937	**0.002**
DQOL_Dissatisfaction	42.25 (9.11)	29.50 (4.81)	**0.012**	43.83 (9.75)	42.40 (8.84)	0.070	0.607	**0.001**
DQOL_Impact	41.17 (7.57)	30.87 (4.51)	**0.012**	40.76 (10.16)	40.36 (9.77)	0.184	0.659	**0.008**
DQOL_Social worry	15.74 (4.94)	10.87 (3.09)	**0.011**	16.56 (6.92)	14.35 (5.83)	**0.038**	0.777	0.096
DQOL_Diabetes worry	11.40 (3.16)	7.75 (1.48)	0.051	11.73 (3.03)	10.65 (3.28)	**0.022**	0.761	**0.009**
FH-15	35.77 (11.15)	29.87 (9.23)	**0.018**	39.30(13.10)	37.10 (12.98)	0.191	0.275	0.212
STAI-S	62.94 (27.82)	23.75 (30.36)	0.050	54.46 (26.62)	48.95 (30.09)	0.076	0.137	**0.039**
STAI-T	74.23 (25.07)	77.76 (20.10)	**0.012**	77.76 (20.10)	66.50 (27.07)	**0.006**	0.937	**0.004**
DDS_total	2.61(0.94)	1.48 (0.40)	**0.012**	2.44 (0.95)	2.36 (1.16)	0.193	0.407	**0.015**
DDS_emotional	3.10 (1.29)	1.65 (0.77)	**0.017**	3.20 (1.39)	2.91 (1.38)	0.064	0.879	**0.018**
DDS_physical	1.82 (1.00)	1.09 (0.18)	**0.027**	1.55 (0.88)	1.81 (1.33)	0.332	0.111	0.157
DDS_regimen	2.88 (1.12)	1.75 (0.58)	**0.041**	2.56 (1.11)	2.52 (1.38)	0.176	0.243	0.295
DDS_interpersonal	2.40 (1.18)	1.29 (0.27)	**0.018**	2.17 (1.08)	1.90 (1.15)	0.240	0.454	0.239
SCI-R	61.56 (14.29)	76.25 (8.01)	**0.027**	64.94 (10.46)	62.62 (8.35)	0.200	0.436	**0.002**
HbA1c (%)	8.22 (1.51)	7.90 (1.40)	1.000	8.31 (1.48)	8.46 (1.60)	0.968	0.657	0.576

Mean (Standard deviation).

The bold values denote statistical significance at P ≤ 0.05 level.

At the end of the treatment program, the subjects were reassessed. **Table 2** displays the baseline and post-treatment values. In the TG, only those who completed the treatment program were evaluated (eight participants). In the CG, twenty participants were evaluated.

The participants in the TG exhibited a statistically significant improvement in scores on the BDI-FS, the total DQOL and its subscales Dissatisfaction, Impact, and Social worry with the decrease in their scores with respect to the baseline evaluation, although there are no significant differences in the Diabetes worry subscale. In addition, there was a statistically significant improvement in scores on the FH-15, STAI-T (not however in STAI-S), the total DDS and its subscales Emotional, Physical, Regimen, and Interpersonal. Significant differences were also found in the scores on the SCI-R, with an increase in the percentage of treatment adherence at the end of the program. No significant differences are found in HbA1c.

The participants in the CG presented differences on the BDI-FS, the DQOL subscales Social worry and Diabetes worry, and on the STAI-T, decreasing the scores in these scales in the post-treatment evaluation. However, they do not present significant differences in total DQOL and the Dissatisfaction and Impact subscales, in the FH-15, in STAI-S, in the total DDS and its subscales, in the SCI-R and in HbA1c.

Regarding the differences between the groups at the end of the program, better scores were found in the TG on the BDI-FS, the total DQOL, DQOL Dissatisfaction, DQOL Impact, DQOL Diabetes worry, STAI-S and STAI-T, total DDS and DDS emotional, with the decrease in the score of these scales, and on the SCI-R, increasing the score and, therefore, the adherence of these people. No differences were found in the scores between groups in the post-treatment in the DQOL Diabetes worry subscale, in FH-15, in the subscales of the DDS psysical, regimen and interpersonal and in HbA1c.

In addition, in the post-treatment evaluation, the number of cases of diagnosis of major depressive episode in the TG decreased to zero subjects, while in the CG this decreased to two.

**Table 3** provides the percentages of change in the variables studied, comparing the results between the two groups. According to the results, the participants in the TG presented a significantly greater change with respect to the CG in the variables BDI-FS, DQOL, DQOL Dissatisfaction, DQOL Impact, DQOL Social worry, STAI-T, the total DDS and in its Emotional, Physical and Interpersonal subscales, and in the SCI-R. However, there is no change in the DQOL Diabetes worry subscale, in FH-15, STAI-S, in the DDS regimen subscale and in HbA1c.

**Table 3:** Percentage change in study variables.

Variables (scores)	Treatment Group (n=8)	Control Group (n=20)	p
BDI-FS	88.54 (23.96)	-1.29 (11.05)	**0.002**
DQOL_total	26.32 (5.59)	5.95 (13.47)	**≤ 0.001**
DQOL_Dissatisfaction	25.43(7.37)	4.17 (14.57)	**≤ 0.001**
DQOL_Impact	23.63 (9.98)	3.83 (16.76)	**0.004**
DQOL_Social Worry	31.87 (10.13)	7.90 (43.62)	**0.030**
DQOL_Diabetes Worry	22.39 (25.96)	9.56 (19.50)	0.108
FH-15	22.62 (22.40)	4.38 (20.29)	0.093
STAI-S	31.79 (105.96)	9.21 (61.69)	0.093
STAI-T	66.51 (32.55)	19.73 (28.75)	**0.003**
DDS_total	35.24(19.46)	6.61 (29.24)	**0.022**
DDS_emotional	87.37 (4.52)	82.23 (5.79)	**0.037**
DDS_physical	82.64 (5.49)	70.42 (14.58)	**0.006**
DDS_regimen	85.01 (5.81)	81.55 (6.90)	0.387
DDS_interpersonal	80.40 (8.01)	68.43 (13.74)	**0.020**
SCI-R	-13.94 (20.57)	3.81 (13.90)	**0.034**
HbA1c (%)	0.80 (11.63)	-1.29 (11.05)	0.832

Mean (Standard deviation).

The bold values denote statistical significance at P ≤ 0.05 level.

## Discussion and conclusions

This study assessed the efficacy of the Internet-based CBT program for the treatment of mild-moderate depressive symptomatology in individuals with type 1 diabetes (WEB_TDDI1 study). This program comprises nine weekly online sessions (30). The platform was designed to serve as a convenient and accessible tool for people with type 1 diabetes to treat their depressive symptomatology. Previous studies support the positive results of these programs in people with diabetes (10–14). Thus, when we began the program evaluation, we hypothesized that the program would reduce symptomatology and that this improvement would be reflected in the other psychological variables evaluated.

The results were positive, and the program was effective in reducing depressive symptomatology, a finding consistent with earlier studies (18–27). Regarding diabetes-related distress, the second outcome analyzed in the aforementioned studies, the treatment program reduced diabetes-related distress, with positive results both on the general scale and on the four subscales of distress (Emotional, Physical, Treatment Regimen, and Interpersonal).

With respect to anxiety, Newby et al. (25) and Clarke et al. (26) reported improvements, with a moderate effect in the case of Clarke (26). In our study, we found a significant decrease in trait anxiety scores, although the causes of the lack of change in state anxiety should be investigated. Concerning quality of life, this has only been examined in one of the studies that developed web based interventions. Newby et al. (25) analyzed quality of life with the general SF-12 scale, describing improved scores on the mental well-being scale but not on the physical well-being scale. In our study, we chose to use the DQOL scale because it is specific to diabetes. We found positive results with an increase in general quality of life and on the Dissatisfaction, Impact, and Social worry subscales. This program was also shown to be effective in reducing fear of hypoglycemia, a negative variable in diabetes care due to its effect on metabolic control and therefore on patient health (40). In addition, the participants who completed the treatment program had increased adherence to diabetes treatment, a variable that is decreased in individuals with diabetes (5) and which is essential for proper diabetes care and for the prevention of complications associated with the disease.

On the other hand, although the people who have completed the TG have presented improvements in depressive symptoms, quality of life, anxiety, distress and adherence to treatment with respect to the participants of the CG, in some variables (the quality-of-life subscale social worry, fear of hypoglycemia and physical, treatment regimen and interpersonal distress) no significant differences have been found despite improving their scores with respect to the CG. It is possible that the passage of time and being involved in a study could have caused an improvement in these areas in the CG. The sample size could also influence the fact that, despite obtaining better scores in the TG, no significant differences were found.

The data obtained for glycemic control, analyzed with the glycosylated hemoglobin (HbA1c) test, are consistent with previous reviews, which indicate that online treatment is not effective for improving glycemic control, compared to other interventions such as pharmacological therapy, group therapy, and psychotherapy (8, 17). However, it is reasonable to expect a decrease in this variable since the treatment resulted in an improvement in depressive symptoms associated with glycemic control, and therefore a decrease in HbA1c levels would also be anticipated (42, 43). It is possible that the time period between the baseline evaluation and the post-treatment evaluation was not sufficient to find changes in HbA1c. Perhaps other measures of glycemic control should be explored, such as those provided by continuous glucose monitoring or more sensitive real-time continuous glucose readings, which could shed light on the possible short-term effect on glycemic control. It would be advisable to include more sensitive measures of glycemic control facilitated by new technologies in future studies.

Although the results of the study have generally been positive, adherence to the program has been low. In this study, only the evaluations of those people who have completed the nine sessions of the program have been included in the analyses. In order to complete each session and be able to carry out the next session, the participants had to carry out the appropriate task. In this sense, it has been a demanding program, which implied a high involvement and motivation of the participants. The main reasons for leaving the program were: not having time due to work, family problems, having problems with the use of computers/technology or worsening symptoms and the need to refer to Mental Health. These reasons suggest that, although the technology is effective, it is not a universal treatment, but specific for people with specific characteristics, such as a specific profile of motivation and interest in treatment and with knowledge of new technologies. In addition, although in this study the participants did not have to be undergoing psychological or psychiatric treatment to assess the “pure” effect of the intervention. For use in clinical practice, it can be considered as a complement to the usual treatment for these problems. These results could imply that, in line with the diabetes treatments used in this population that require personalization and precision, the use of Internet-based programs for the treatment of depressive symptoms is a treatment option that can be considered for people with type 1 diabetes with specific characteristics and needs. On the other hand, it could also be interesting to study possible changes in the format of the platform that could make it simpler and more useful in the day-to-day life of the user.

One of the main strengths of this study is that we attempted to determine the “pure” effect of the intervention in individuals with type 1 diabetes. To do this, the subjects selected were not undergoing psychological or psychiatric treatment at the time. It would perhaps be advisable for future evaluations of the program to include individuals receiving another type of treatment for their depressive symptomatology in order to determine the effect of addition of this treatment to conventional care compared toits addition to online therapy.

Furthermore, positive results were found in other variables related to diabetes management (distress, anxiety, fear of hypoglycemia, and quality of life). Adherence to diabetes treatment also improved.

The main problem encountered in this study was treatment adherence. Despite reminder emails and phone calls, the number of people evaluated at the beginning of the program decreased compared to those who successfully completed the nine sessions. Furthermore, the evaluation was only carried out on those people who completed the 9 sessions, so the sample and its possible follow-up were considerably reduced. The study of mechanisms to increase adherence and the profile of people who adhere to this program seems to be the next step to follow, with the goal of offering this program to those people who could derive true benefit from it.

Future work should aim to study the variables associated with adherence as well as the profiles of subjects who may benefit most from this type of treatment. In addition, the effect of this program should be analyzed in a larger sample, including individuals undergoing other types of psychological or psychiatric treatment. On the other hand, it would also be recommendable to study possible changes in the format adapted to user interest.

The objective of this study has been assessed the efficacy of the Internet-based CBT program for the treatment of mild-moderate depressive symptomatology in individuals with type 1 diabetes (WEB_TDDI1 study). In this sense, it can be concluded that in the population that has completed the treatment the Internet-based CBT program for depressive symptomatology in type 1 diabetes has been effective for the treatment of mild-moderate depressive symptomatology. In addition, this has been a suitable program for addressing other important areas in diabetes management such as diabetes-related distress, anxiety, fear of hypoglycemia, and quality of life to improve treatment adherence in people with type 1 diabetes and depressive symptoms. Future research will help to establish profiles of program beneficiaries and possible improvements in the program to improve adherence to treatment.

## Data availability statement

The datasets generated for this study are available on request to the corresponding author.

## Ethics statement

This study was approved by the Provincial Research Ethics Committee of Malaga (approval number: 1216/PIA14), Spain (December 20, 2016). The studies were conducted in accordance with the local legislation and institutional requirements. The participants provided their written informed consent to participate in this study.

## Author contributions

MC and MTA were responsible for the conceptualization, funding acquisition, web treatment design, project administration, data analysis, supervision, and revision of the manuscript. MC was responsible for data collection, database creation, interpretation of results, and manuscript preparation. MSR and JLP were responsible, together with MC, for subject recruitment and data collection. All authors contributed to the article and approved the submitted version.
